# Machine learning-based algorithms for the prediction of 90-day survival in patients with liver failure receiving artificial liver therapy

**DOI:** 10.3389/fphys.2025.1687860

**Published:** 2025-10-27

**Authors:** Bo Deng, Chengzhi Bai, Huaqian Xu, Xue Zhang, Ying Deng

**Affiliations:** ^1^ Department of Gastroenterology, The General Hospital of Western Theater Command, Chengdu, Sichuan, China; ^2^ Graduate School of Chengdu Medical University, Chengdu, Sichuan, China; ^3^ Integrated Care Management Center, Institute of Respiratory Health, West China Hospital, Sichuan University, Chengdu, China

**Keywords:** liver failure, artificial liver therapy, survival, machine learning, predictive value

## Abstract

**Background:**

Liver failure is associated with high short-term mortality, and the predictive value of clinical factors for patients undergoing artificial liver therapy is uncertain. We aim to develop prognostic models using several machine learning algorithms to predict 90-day survival in patients with liver failure undergoing artificial liver therapy.

**Methods:**

We retrospectively enrolled hospitalized patients with liver failure who received artificial liver therapy in our center between December 2017 and December 2021. Prognostic characteristics were chosen by the least absolute shrinkage and selection operator (LASSO) regression and independent predictors by stepwise logistic regression analysis. Five machine learning algorithms—logistic regression (LR), random forest (RF), support vector machine (SVM), eXtreme Gradient Boosting (XGBoost), and k-nearest neighbor (KNN)—were used to build and validate models to predict 90-day survival following Artificial liver support systems. The model performance was assessed by the area under the receiver operating characteristic curve (AUC), accuracy, sensitivity, specificity, positive predictive value, and negative predictive value.

**Results:**

A total of 197 patients were included in this study. LASSO regression, based on patient admission data, identified the top 15 prognostic features, and stepwise LR analysis determined that the age, direct bilirubin, retinol, alpha-fetoprotein, and thrombin time were independent predictors. Among the five machine learning models, LR achieved the highest predictive performance with an AUC of 0.884 and accuracy of 75.0%, followed by RF (AUC = 0.797), KNN (AUC = 0.788), XGBoost (AUC = 0.769), and SVM (AUC = 0.732). The predictive performance of LR models based on longitudinal data using patient characteristics from the day before treatment had an AUC of 0.869, and from the day after treatment, it had an AUC of 0.859.

**Conclusion:**

Machine learning models showed promising performance in predicting 90-day survival in liver failure patients receiving artificial liver support therapy, potentially supporting individualized prognostic assessment.

## Introduction

Liver failure is a life-threatening syndrome characterized by rapid liver dysfunction, severe coagulopathy, and multi-organ failure, presenting as acute liver failure (ALF), acute-on-chronic liver failure (ACLF), or chronic liver failure (CLF) depending on the etiology and disease course (Perez Ruiz de Garibay et al., 2022; Wang X et al., 2024). Liver failure significantly contributes to augmented morbidity and mortality worldwide, accounting for more than 2 million deaths yearly, with deaths due to liver disease increasing by 50% over the last 3 decades and predicted to double over the next 20 years (GBD, 2017 Cirrhosis Collaborators, 2020; Zwirner et al., 2024). Specific physiological defects contribute to disease progression in the varying forms of liver failure. In ALF, failure of the liver to eliminate toxins from the body causes systemic inflammation, coagulation abnormalities, and kidney damage, with the mortality greater than 50% (Tujios et al., 2022). CLF, following cirrhosis of the liver, causes slow deterioration of the liver’s capabilities, and it is accountable for more than 1.5 million deaths each year, with a 1-year mortality of 40%–60% at the late stages of the disease (Wu et al., 2024). In ACLF, failure of the liver to maintain physiological control causes multiple organ failure, with greater than 90-day mortality of 50% or greater (European Association for the Study of the Liver, 2023). Thus, it is important to develop predictive models for the prognosis of liver failure to prevent delayed progression, limit complications, and enhance the overall management.

Artificial liver support systems (ALSSs) have been utilized to manage liver failure. They are extracorporeal therapies utilized to eliminate toxins, restore plasma components, and temporarily support the liver, facilitating liver regeneration or allowing time to wait for liver transplantation (Lan et al., 2025). Among these, plasma exchange (PE) and its combination with double plasma molecular adsorption system (DPMAS) are commonly used. PE removes bilirubin, ammonia, and inflammatory mediators, and DPMAS specifically eliminates protein-bound toxins and cytokines, minimizing donor plasma requirements (Tan et al., 2020). Clinical trials have attested that PE markedly attenuates the systemic inflammatory response syndrome (SIRS) in ALF patients and enhances their survival rates (Maiwall et al., 2020). Furthermore, PE in combination with DPMAS improves the coagulation function, decreases organ failure scores, and enhances short-term survival, especially in hepatitis B virus (HBV)-related ACLF patients (Guo et al., 2020).

Previous studies have shown that the development and progression of hepatic failure are regulated by a multitude of physiological mechanisms and clinical variables. Hepatic encephalopathy (HE) results from the accumulation of the body’s metabolic byproducts, leading to neurological malfunction and causing systemic inflammation that contributes to worsening hepatic damage. Low pre-albumin levels are indicative of impaired hepatic synthetic capabilities and malnutrition, signifying a decline in liver physiological capabilities, while higher levels of serum creatinine coincide with acute kidney injury (AKI), additionally worsening liver failure (Figueira et al., 2021; Zhang et al., 2023). In addition, studies have shown that systemic inflammation is responsible for organ functional failure, while renal functional failure also impairs the processes of detoxification. These factors are responsible for multiorgan failure and accelerate liver damage, which are independent predictive factors of in-hospital mortality among patients with ALF (Thuluvath et al., 2021; Tong et al., 2019). Although the usual predictive models such as MELD and the Child–Pugh scoring system are widely practiced in clinical scenarios, they are unable to reflect the complexities of such physiological processes effectively and, hence, have limited predictive capabilities, specifically within situations of complicated liver failure. Recently, machine learning models, such as decision trees, random forest (RF), support vector machine (SVM), and deep learning, have been demonstrated to have superior predictive power in short- and long-term outcome prediction (Panackel et al., 2024; Qiu et al., 2024). Research on prognostic prediction after ALSS is sparse, especially so with machine learning algorithms.

Because of the high mortality of liver failure, the restrictedness of conventional prognostic scores, and the promise of machine learning in precision medicine, we sought to establish and validate prognostic models to predict 90-day survival for liver failure patients undergoing ALSS. With LASSO-selected variables and a variety of machine learning algorithms, we sought to determine the crucial prognostic factors and compare their predictive value for facilitating personalized treatment planning.

## Methods

### Study design and patients

This was a retrospective cohort study, and it was carried out after being approved by the Ethics Review Committee of the General Hospital of Western Theater Command (No. 2020ky005). The study was in accordance with the principles of the Helsinki Declaration, conformed to the Strengthening the Reporting of Observational Studies in Epidemiology (STROBE) guidelines, and was in compliance with all relevant national laws. Due to the retrospective, anonymous nature of the study and its low risk, requirement of informed consent was waived.

We chose patients with liver failure who underwent artificial liver therapy in our hospital between December 2017 and December 2021. The inclusion criteria were as follows: 1) age >18 years old; 2) clinically diagnosed with liver failure; 3) undergoing artificial liver treatment (PE or PE plus DPMAS); 4) integrity of clinical data. The exclusion criteria were as follows: 1) autoimmune liver disease, drug-induced hepatitis, alcoholic liver injury, hepatocellular carcinoma, or other liver malignancies; 2) combined with severe heart, lung, or kidney disease, other cancers, or other serious diseases influencing the prognosis; 3) severe mental or cognitive illness or pregnant women; 4) lack of follow-up data.

### Definition of liver failure

Liver failure is defined as a syndrome resulting from severe liver damage caused by numerous factors that cause prominent decompensation or abundant dysfunction of its physiological functions such as detoxification, biotransformation, metabolism, and synthesis. Clinically, it manifests primarily as a syndrome of hepatorenal syndrome, jaundice, coagulation disorders, hepatic encephalopathy, and ascites (Liver Failure and Artificial Liver Group, 2019). ALF is a clinical syndrome that results in the sudden onset of liver failure in 2 weeks in the form of grade II or higher HE (under grade IV classification); fast progressive jaundice with serum TBIL of at least 10 times the upper limit of normal (ULN) or a daily rise of at least 17.1 μmol/L; bleeding tendencies with PTA of 40% or less (or INR of 1.5 or more); extreme exhaustion; marked anorexia; abdominal distension, nausea, vomiting, and other extreme gastrointestinal symptoms; and progressive shrinking of the liver. CLF is characterized as slow liver failure and decompensation based on cirrhosis, occurring in the form of raised TBIL (usually <10 times ULN), low albumin, low platelet count, and PTA of 40% or less (or INR of 1.5 or more), along with refractory ascites/portal hypertension and HE. ACLF is a syndrome of acute/subacute liver decompensation based on pre-existing chronic liver disease, occurring in the form of rapidly progressive jaundice, serum TBIL of at least 10 times ULN (or a daily rise of at least 17.1 μmol/L), and bleeding with PTA of 40% or less (or INR of 1.5 or more).

### Artificial liver therapy

For PE therapy, the femoral vein is catheterized with a double-lumen catheter to establish extracorporeal circulation, connecting to the artificial liver support system. The plasma separator removes 2,500 mL–3,000 mL of fresh plasma, and an equivalent volume of fresh frozen plasma is substituted. The blood flow rate is 100 mL/min, with a plasma exchange flow rate of 20 mL/min. Each session lasts 2 h–3 h and is repeated every 3 to 5 days. PE is also used in patients with severe HE, refractory hyperbilirubinemia, hepatorenal syndrome, and toxin-induced liver damage, particularly in the presence of coagulopathy, severe jaundice, or multi-organ failure (Agrawal et al., 2025; Yuan et al., 2018). PE in combination with DPMAS therapy is particularly used in patients with toxin-induced liver damage, refractory hyperbilirubinemia, or in patients needing to remove protein-bound toxins and cytokines, offering premium liver support along with effective detoxification (Yao et al., 2019). In PE + DPMAS, separated plasma is subjected to a bilirubin adsorber and blood perfusion unit under dual adsorption, which eliminates bulk molecule toxins, cytokines, bilirubin, and numerous detrimental compounds, further enhancing liver performance. The duration of each session is 3 h–4 h, repeated every 3 to 5 days.

### Data collection

We retrieved two main categories of information from the de-identified patient records, including data from three key time points: at the time of admission, the day before ALSS treatment, and the day after ALSS treatment. Clinical data included patient demographics such as age, sex, type of liver failure, number of complications, hepatitis B virus (HBV) positivity, and presence of cirrhosis. The complications include HE ascites, AKI, variceal bleeding, electrolyte imbalance, and spontaneous bacterial peritonitis (SBP). HE is confirmed based on the West Haven criteria. The presence of ascites is confirmed by ultrasound assessment. AKI is identified based on the serum creatinine levels, with a rise of ≥0.3 mg/dL being qualified as AKI. Variceal bleeding is confirmed by upper gastrointestinal endoscopy. Electrolyte imbalance is confirmed by hematological examination. Spontaneous bacterial peritonitis is confirmed by culturing the ascitic fluid. Meanwhile, laboratory test results include alpha-fetoprotein (AFP), albumin, pre-albumin, total bilirubin (TBIL), direct bilirubin (DBIL), indirect bilirubin (IBIL), alanine aminotransferase (ALT), aspartate aminotransferase (AST), γ-glutamyl transferase (γGGT), alkaline phosphatase (ALP), total bile acid (TBA), cholinesterase, retinol, urea, creatinine, cystatin C, endogenous creatinine clearance, uric acid (UA), prothrombin time (PT), INR, fibrinogen, activated partial thromboplastin time (APTT), thrombin time (TT), white blood cell (WBC) count, red blood cell (RBC) count, hemoglobin, platelet count, neutrophil count, lymphocyte count, monocyte count, potassium, sodium, chloride, and C-reactive protein (CRP). The outcome measure is the 90-day survival rate, and the patient survival status is confirmed by an interview over the phone and home visit. Follow-up time points are at 90 days post-discharge.

### Statistical analysis

Statistical analysis was conducted with Python 3.13. The variables were all classified based on their type. Continuous variables that were normally distributed were presented as mean ± standard deviation (SD), and non-normally distributed continuous variables were presented as the median (interquartile range, Q1–Q3). These were compared across groups using Welch’s t-test or the Mann–Whitney U test, accordingly. Categorical variables were presented as counts and percentages and compared using the Chi-square test or Fisher’s exact test, accordingly. A two-tailed p-value <0.05 was regarded as statistically significant. Power analysis was conducted to assess the statistical power for detecting the survival differences among different liver failure types.

Feature selection was carried out using the least absolute shrinkage and selection operator (LASSO) regression approach by applying L1 regularization along with 5-fold cross-validation in order to select the best regularization parameter (λ), thus minimizing the possibility of overfitting. Stepwise LR was performed, where statistically insignificant variables were progressively selected or removed to identify the optimal combination of the variables. The coefficients (Coef), standard errors, z-values, p-values, and 95% confidence intervals (CI) of each variable were estimated to identify the independent predictors of survival. Machine learning models such as logistic regression (LR), RF, SVM, eXtreme Gradient Boosting (XGBoost), and K-nearest neighbor (KNN) were then utilized for developing risk prediction models based on these independent risk factors. The dataset was then randomly divided into a training set (70%) and a validation set (30%), and hyperparameter tuning was carried out in the training set using grid search with cross-validation. Regularization and early stopping techniques were utilized to avoid overfitting. The performance of the models was assessed using the single validation set, with evaluation metrics including the area under the ROC curve (AUC) and its 95% CI, accuracy, sensitivity, specificity, positive predictive value (PPV), negative predictive value (NPV), and F1 score. Shapley additive explanation (SHAP) was utilized in the interpretation of model predictions and in the identification of the most influential contributors toward survival. Based on the MELD and Child–Pugh scores, we constructed prediction models to compare the performance of machine learning models with that of traditional models. We also conducted a longitudinal data analysis based on patient characteristics measured on the day before treatment and the day after treatment.

## Results

### Characteristics of the participants at the time of admission

**FIGURE 1:**
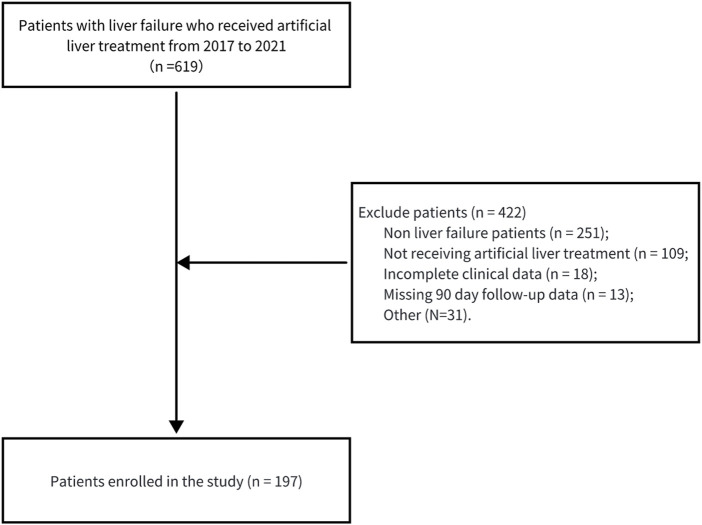
Flow diagram depicting the participant selection process.

Table 1 shows the summary of baseline characteristics of the patients at the time of admission involved in this study. This study included 197 subjects (Figure 1). The survival group cohort included 154 patients, while the non-survival group included 43 patients. The male gender was predominant in both cohorts (83.12% vs. 79.07%), and no statistically significant difference was found between the survival and non-survival groups (p = 0.70). The survival group members were significantly younger (46.88 ± 11.79 years) than those in the non-survival group (55.02 ± 10.73 years), with a statistically significant difference (p < 0.05). The largest percentage of patients in both groups were diagnosed with ACLF (72.73% vs. 62.79%), and most individuals in both groups received PE treatment (55.19% vs. 58.14%). Additionally, the non-survival group had higher levels of TBIL, DBIL, and IBIL, while the survival group had significantly higher levels of albumin and pre-albumin (p < 0.05). In terms of coagulation function, the non-survival group had significantly higher PT, INR, and APTT, and the fibrinogen level was significantly lower. In addition, the non-survival group had lower platelet and RBC counts (p < 0.05).

**TABLE 1:** Characteristics of the included liver failure patients.

Variable	Survival cohort n = 154	Non-survival cohort n = 43	p-value
Age, year	46.88 ± 11.79	55.02 ± 10.73	<0.01
Gender			0.70
Male	128 (83.12%)	34 (79.07%)	
Female	26 (16.88%)	9 (20.93%)	
Type of liver failure			<0.01
Acute-on-chronic	112 (72.73%)	27 (62.79%)	
Acute	30 (19.48%)	3 (6.98%)	
Chronic	12 (7.79%)	13 (30.23%)	
Complications			0.28
1	107 (69.48%)	25 (58.14%)	
2	35 (22.73%)	13 (30.23%)	
3	9 (5.84%)	5 (11.63%)	
4	3 (1.95%)	0 (0.00%)	
Hepatitis B	110 (71.43%)	32 (74.42%)	0.84
Cirrhosis	70 (45.45%)	23 (53.49%)	0.45
MELD score	12.12 9.64, 15.36)	16.47 (13.05, 18.72)	<0.01
Child–Pugh score			<0.01
A	78 (50.65%)	30 (69.77%)	
B	60 (39.61%)	7 (16.28%)	
C	14 (9.74)	5 (13.95%)	
Artificial liver treatment			0.21
PE	85 (55.19%)	25 (58.14%)	
PE + DPMAS	69 (44.81%)	18 (41.86%)	
Artificial liver therapy sessions	3 (2, 4)	3 (2, 4)	0.14
AFP, μg/L	149.77 ± 301.30	49.34 ± 65.43	0.03
Albumin, g/L	34.75 ± 5.16	31.69 ± 3.80	<0.01
Pre-albumin, mg/L	68.33 ± 37.94	50.15 ± 18.30	<0.01
TBIL, μmol/L	314.96 ± 136.43	401.94 ± 141.21	<0.01
DBIL, μmol/L	198.35 ± 96.80	253.48 ± 106.64	<0.01
IBIL, μmol/L	116.62 ± 50.89	148.45 ± 53.85	<0.01
ALT, U/L	1020.02 ± 1024.66	677.62 ± 745.69	0.04
AST, U/L	739.22 ± 760.61	620.17 ± 782.60	0.37
γGGT, U/L	202.06 ± 392.42	156.69 ± 189.08	0.46
ALP, U/L	198.52 ± 145.36	204.22 ± 75.72	0.81
TBA, μmol/L	220.92 ± 92.74	232.04 ± 89.55	0.48
Cholinesterase, U/mL	4.32 ± 1.73	3.61 ± 1.44	0.02
Retinol, μg/dL	16.96 ± 9.02	14.89 ± 3.97	0.15
Urea, mmol/L	4.60 ± 2.43	5.12 ± 2.59	0.22
Creatinine, mg/dL	0.81 ± 0.46	0.85 ± 0.39	0.62
Cystatin C, mg/L	1.09 ± 0.34	1.18 ± 0.43	0.13
ECC, mL/min	83.47 ± 20.41	77.28 ± 19.47	0.08
Uric acid, μmol/L	211.41 ± 91.40	192.71 ± 100.52	0.25
Prothrombin time, s	17.97 ± 5.83	21.26 ± 6.66	<0.01
INR	1.59 ± 0.54	1.88 ± 0.60	0.01
Fibrinogen, g/L	1.80 ± 0.92	1.47 ± 0.50	0.03
APTT, s	41.85 ± 9.98	47.34 ± 10.27	<0.01
TT, s	22.79 ± 2.95	24.79 ± 3.91	<0.01
WBC count, 10^3^/μL	6.51 ± 2.94	6.52 ± 2.41	0.98
RBC count, 10^6^/μL	4.48 ± 0.81	4.12 ± 0.83	0.01
Hemoglobin, g/L	137.43 ± 23.17	130.33 ± 23.32	0.08
Platelets, 10^3^/μL	144.52 ± 132.07	103.40 ± 45.58	0.05
Neutrophils, 10^9^/L	4.72 ± 2.51	4.89 ± 1.99	0.69
Lymphocytes, 10^9^/L	1.15 ± 0.57	0.98 ± 0.46	0.08
Monocytes, 10^9^/L	0.57 ± 0.28	0.54 ± 0.31	0.55
Potassium, mmol/L	3.85 ± 0.44	3.83 ± 0.58	0.79
Sodium, mEq/L	142.93 ± 80.91	134.42 ± 4.96	0.49
Chloride, mmol/L	100.01 ± 4.67	99.29 ± 4.68	0.37
CRP, mg/L	14.41 ± 11.66	17.35 ± 8.00	0.12

PE, plasma exchange; DPMAS, double plasma molecular adsorption system; AFP, alpha-fetoprotein; TBIL, total bilirubin; DBIL, direct bilirubin; IBIL, intermediate bilirubin; ALT, alanine aminotransferase; AST, aspartate aminotransferase; γGGT, γ-glutamyl transferase; ALP, alkaline phosphatase; TBA, total bile acids; ECC, endogenous creatinine clearance; INR, international normalized ratio; APTT, activated partial thromboplastin time; TT, thrombin time; WBC, white blood cell; RBC, red blood cell; CRP, C-reactive protein; MELD, model for end-stage liver disease.

### Survival rate

**FIGURE 2:**
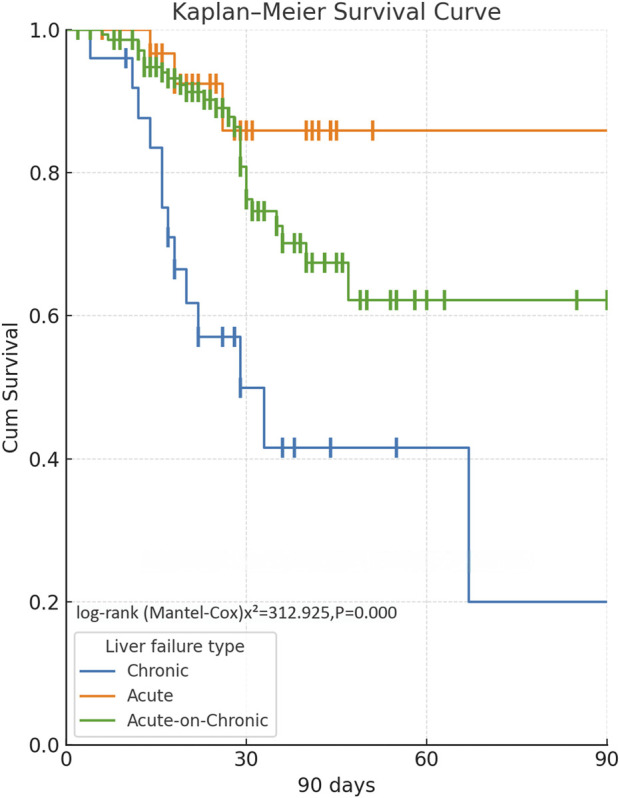
Comparison of 90-day survival curves for patients with different types of liver failure.

At the 90-day follow-up, 85 of 112 patients with ACLF survived (75.89%), 30 of 33 patients with ALF survived (90.91%), and 12 of 25 patients with CLF survived (48.00%). The survival curve reveals that patients with ACLF have the best survival outcomes, with most patients surviving the 90-day duration. Patients with ALF also have relatively favorable survival, but a proportion of patients still die. Patients with CLF have the worst survival, with the curve declining sharply, suggesting that the majority of the patients die within 90 days. The log-rank test p-value of 0.00 suggests that the differences in survival among the types of liver failure are statistically significant (Figure 2). The power analysis results showed that the effect size between ALF and CLF was 0.429 with a power of 0.52, that between ACLF and ALF was 0.103 with a power of 0.08, and that between ACLF and CLF was 0.326 with a power of 0.45.

### Feature screening results based on patients’ admission data

**FIGURE 3:**
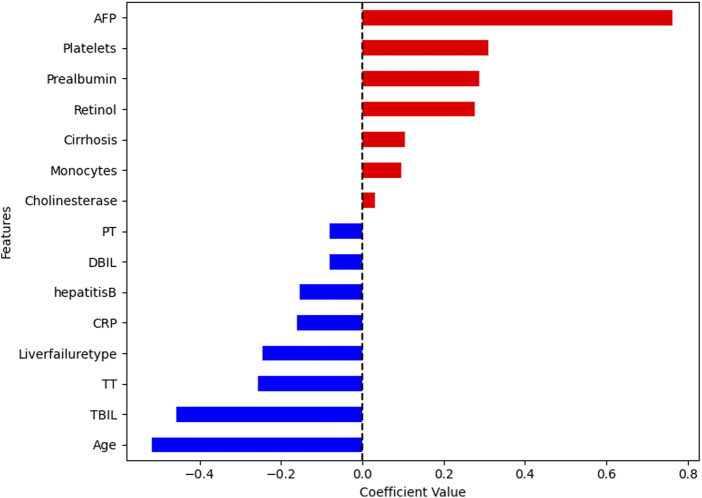
LASSO regression coefficients of the selected features for predicting 90-day survival in patients with liver failure based on patient admission data.

Within the context of LASSO regression, the optimal λ identified using cross-validation was log(λ) = 0.73, which retained the top 15 features with coefficients (Supplementary Figure S1). The features that were retained included age, AFP, HVB, liver failure type, cirrhosis, DBIL, TBIL, CRP, retinol, pre-albumin, platelets, PT, cholinesterase, TT, and monocytes (Figure 3). Stepwise LR using the features selected by LASSO indicated that the age (Coef = −0.064, 95% CI: −0.103 to 0.81, and p = 0.001), DBIL (Coef = −0.007, 95% CI: −0.011 to −0.013, and p = 0.000), retinol (Coef = 0.091, 95% CI: 0.012 to 0.171, and p = 0.024), AFP (Coef = 0.008, 95% CI: 0.002 to 0.003, and p = 0.007), and TT (Coef = −0.185, 95% CI: −0.318 to −0.051, and p = 0.007) were independent predictors (Table 2).

**TABLE 2:** Result of the stepwise logistic regression analysis.

Variable	Coefficient	Standard error	95% CI	z-value	p-value
Age	−0.064	0.020	−0.103 to −0.026	−3.259	0.001
DBIL	−0.069	0.002	−0.011 to −0.003	−3.523	0.000
Retinol	0.091	0.040	0.012 to 0.171	2.256	0.024
AFP	0.008	0.003	0.002 to 0.013	2.700	0.007
Thrombin time	−0.185	0.068	−0.318 to −0.051	−2.701	0.007

CI, confidence interval; DBIL, direct bilirubin; AFP, alpha-fetoprotein.

### Comparison of different prediction models

**FIGURE 4:**
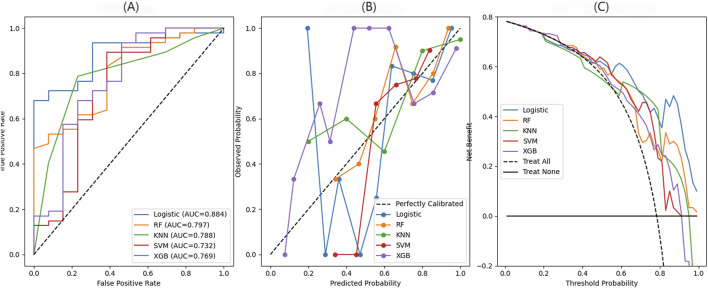
Comparison of 90-day survival prediction performance in liver failure patients across different machine learning models. **(A)** Comparison of ROC curves for different machine learning models illustrating the performance in predicting 90-day survival. **(B)** Decision curve analysis for different machine learning models evaluating the net clinical benefit of each model at varying threshold probabilities. **(C)** Calibration curve for different machine learning models showing the agreement between the predicted survival probabilities and the observed outcomes.

On the basis of these independent risk factors, we constructed predictive models with various machine learning algorithms to predict the 90-day survival rate of liver failure patients following artificial liver treatment. The LR model showed optimal predictive power; the model had an AUC of 0.884 (0.786–0.960) and accuracy of 75.0%. The other models also showed good predictive power, with the RF model having an AUC of 0.797 (0.663–0.914), the KNN model having an AUC of 0.788 (0.642–0.907), the XGBoost model having an AUC of 0.769 (0.585–0.918), and the SVM model having an AUC of 732 (0.527–899) (Figure 4A; Supplementary Table S1). Across the five models, LR showed the most balanced performance with better discrimination, net benefit, and calibration. RF and SVM achieved moderate benefits in the medium probability range but showed decreased performance at higher thresholds. XGBoost exhibited a similar trend to RF and SVM, with moderate benefits but a decline at higher thresholds. KNN was less stable overall, with greater fluctuations and deviations from the ideal calibration line (Figures 4B, C; Supplementary Figure S2). We also established traditional machine learning models by using the MELD score and the Child–Pugh score, and their AUCs were 0.574 and 0.673, respectively; both of them are less than that of the machine learning model (Supplementary Figure S3).

### SHAP-based model interpretability analysis

SHAP analysis findings indicated that AFP was the highest predictor in ascertaining the 90-day survival in patients undergoing artificial liver support therapy. Other significant predictors, such as age, DBIL, TT, and retinol, also had significant contributions in terms of model output (Supplementary Figure S4).

### Assessment of predictive ability using longitudinal data sets

Patient characteristic analysis was also performed on a day before and after treatment. The LASSO and stepwise LR identified significant variables of age (Coef = −0.504), albumin (Coef = 1.17), IBIL (Coef = −0.874), CRP (Coef = −0.673), and PT (Coef = −0.920) before undergoing ALSS; meanwhile, after a day, the significant variables were age (Coef = −0.754), TBIL (Coef = −0.797), PT (Coef = −0.755), and RBC (Coef = 0.738) (Supplementary Table S2). The predictive power of the models based on the data of patients from a day before treatment produced an AUC of 0.869, while data from after a day produced an AUC of 0.859 (Supplementary Table S3; Supplementary Figure S5).

## Discussion

This study examined the 90-day survival rates of patients with liver failure treated by ALSS, demonstrating that survival outcomes varied considerably across different liver failure categories. Patients with ACLF and ALF had relatively good survival rates, while those with CLF had much poorer outcomes. LASSO regression, based on patient admission data, identified the top 15 prognostic features, and stepwise LR analysis revealed that AFP, age, DBIL, retinol, and TT were the independent predictors of 90-day survival. Based on these essential variables, predictive models were established using several machine learning methods, and their performance was compared systematically. Among the five models considered, LR showed the best overall predictive power, outperforming RF, SVM, XGBoost, and KNN in terms of discrimination accuracy and calibration reliability. The SHAP analysis identified AFP as the most influential factor in predicting 90-day survival in patients undergoing artificial liver support therapy. The confusion matrix, decision curve, and calibration plot analyses showed that the LR model accurately classified survivors and non-survivors, offering the greatest clinical benefit and closely aligning predictions with observed outcomes, particularly at high probabilities. The longitudinal analysis based on data from the day before and the day after treatment also demonstrated good predictive performance for the LR model.

The prognostic research of liver failure has gained extensive attention, and earlier studies have primarily targeted general patient populations or etiologies (Li W et al., 2024; Zhu et al., 2024). In recent years, increasing interest has been shown in the prognostic and survival risk factors of patients treated with artificial liver support therapy. A study on HBV–ACLF patients showed that a nomogram based on independent prognostic factors such as age, mid-to-late stage liver failure, HE, upper gastrointestinal bleeding, and the mode of artificial liver therapy (PE + DPMAS) demonstrated good AUC (Wang F et al., 2024). Another study demonstrated that total bilirubin, international normalized ratio (INR), serum creatinine, and age were prognostic factors for the 28-day survival of ACLF patients treated with PE therapy and also obtained good predictive performance (Huang et al., 2019). Du et al. developed the PALS prognostic model based on cirrhosis, TBIL, INR, infection, and hepatic encephalopathy, which accurately predicts the 90-day mortality risk in liver failure patients undergoing PE (Du et al., 2021). Compared to previous studies that used traditional models, adopting a machine learning approach resulted in a moderate increase in the predictive ability (Shi et al., 2024). In terms of applying machine learning models, researchers established an artificial neural network model based on clinical information of HBV–ACLF patients that could predict the mortality risk at 90 days and significantly outperform traditional models (Hou et al., 2020). XGB-CV and decision tree models also markedly outperformed traditional standard models in predicting short-term outcomes among patients with ACLF (Verma et al., 2023). These findings are consistent with our results.

While ALSS can enhance the prognosis of liver failure patients, significant survival disparities still exist among patients, and the determination of independent risk factors is essential (Zhang et al., 2024). Age, DBIL, and TT are crucial physiological parameters that demonstrate liver function and physiological status. With increasing age, the capacity of the liver to regenerate diminishes, and physiological buffers are depleted. Comorbidities and immune dysfunction continue to worsen, lowering the body’s tolerance against circulatory demands and anticoagulation hazards of ALSS and other treatments that could elevate the threat of mortality (Ma et al., 2024; Wu et al., 2018). In case of liver failure, compromised hepatocellular functions impede the clearance of normally secreted bile, leading to an acute elevation of DBIL. Increased DBIL indicates the liver’s compromised detoxifying capacity, aggravated toxin accumulation, systemic inflammation, and hepatocellular injury that results in multi-organ failure (Liang et al., 2022; Xiang et al., 2024). Moreover, an elevated TT signifies an extended coagulation process, which may indicate a disrupted coagulation cascade resulting from the impaired synthesis of coagulation factors in cases of liver failure. This impairment can elevate the risk of hemorrhage, thereby exacerbating the clinical prognosis for individuals with liver failure (Roy et al., 2024).

In the present work, LR also indicated greater values of AUC than other machine learning algorithms. This finding could be explained by the consideration that selected predictors possess approximate linear relationships with survival end-point results consistent with the underlying assumptions of LR. Moreover, on relatively limited clinical data with few predictors, LR will tend to yield more consistent robust performance, such that tree-based and kernel methods are more prone to issues of underfitting or overfitting unless the hyperparameters are optimally fine-tuned. Smooth monotonic probability estimation by LR also permits more stable risk ranking at varying thresholds than stepwise or ill-calibrated probability outputs of ensemble or nonparametric methods (Lynam et al., 2020; Nusinovici et al., 2020). Based on the analysis of the model of LR by SHAP, AFP is the most contributory predictor. Increased AFP levels indicate the damaged regeneration capacity of hepatocytes and the clearance of harmful agents, such that they are indicative of severe liver damage with poor repair capacity of the liver, both of which are consistent with poor prognosis with more severe liver disease pathophysiology (Li C et al., 2024). Retinol exhibits properties that are anti-inflammatory, antioxidant, and immune-regulatory in nature. It has the capacity to mitigate liver damage and enhance hepatic metabolism and regeneration by curbing the excessive activation of hepatic stellate cells and modulating bilirubin metabolism. Specifically, in patients suffering from ACLF and ALF, increased concentrations of retinol may decelerate the progression of liver fibrosis, alleviate hepatic burden, and possibly diminish the mortality risk (Chen et al., 2025; Romeo and Valenti, 2016).

This study’s comparison of 90-day survival across various types of liver failure revealed that ALF and ACLF patients had significantly better survival compared to CLF patients. In patients with ACLF, while there is an underlying chronic liver disease, hepatic function may still recover partially if the acute precipitating factors are effectively controlled early (Li et al., 2023). ALF typically occurs in the setting of previously normal liver function, and their hepatocyte regenerative capability is relatively intact, allowing for better response to active supportive care despite the risk of early rapid deterioration (Ocak, 2023). CLF patients, however, are frequently in a phase of irreversible hepatic parenchymal injury, along with portal hypertension, malnutrition, and various comorbidities. Their hepatocyte regenerative capability is poor, and the benefit of ALSS is minimal, which together may account for the steep decline seen in this group’s survival curve (Saliba et al., 2022). Treatment strategies and monitoring schedules, thus, need to be individualized based on the type of liver failure to ensure the optimization of intervention benefits.

### Limitations

Our study has several limitations. (1) The single-center, retrospective design may introduce selection bias and limit the generalizability of findings. The absence of an external validation cohort also calls for prospective multicenter validation to assess model robustness and clinical applicability. (2) The sample size was inadequate to reveal distinctions among different types of liver failure, particularly within the ALF and ACLF cohorts, and power analysis verified that the study lacked sufficient power, thus constraining its generalizability. (3) The dataset imbalance (154 survivors versus 43 non-survivors) might skew the model toward the predominant class. Although several machine learning algorithms were utilized, methods such as SMOTE or class weighting were not implemented, and the performance was predominantly evaluated using AUC-ROC. (4) A solitary validation set for model assessment, which, despite being computationally efficient and appropriate for extensive datasets, may fail to encompass the full range of variability in model performance. (5) Only baseline data were used for prediction, without considering dynamic changes during treatment. Future research could incorporate longitudinal data to enhance the predictive performance.

## Conclusion

Machine learning algorithms that incorporate significant clinical and laboratory parameters enhanced the precision of 90-day survival prediction in patients with liver failure undergoing artificial liver support therapy, among which LR showed the best performance. Such models can promote personalized treatment strategy and offer more robust evidence for clinical decisions.

## Data Availability

The original contributions presented in the study are included in the article/Supplementary Material, further inquiries can be directed to the corresponding authors.
