# Supplementary material for: Machine learning-based algorithms for the prediction of 90-day survival in patients with liver failure receiving artificial liver therapy
Source: Front Physiol. 2025 Oct 27;16:1687860. doi: 10.3389/fphys.2025.1687860 (PMC12598398; doi:10.3389/fphys.2025.1687860)

**Supplemental files**

**Contents**

**Table**

**Table S1.** Predictive Performance of Machine Learning Models for 90-Day Survival in Liver Failure Patients After Artificial Liver Treatmen...............................................2

**Table S2.** The results of LASSO and stepwise logistic regression analysis based on data from the day before and the day after treatment................................................2

**Table S3.** Predictive performance of logistic regression models for 90-day survival in liver failure patients at different time points..................................................................3

**Figure**

**Figure S1.** Feature selection based on LASSO model. (A) Cross-validation error plot for LASSO regression, showing the mean squared error against log(λ) with the optimal λ values indicated by vertical dashed lines. (B) Plots for LASSO regression coefficients over different values of the penalty parameter...........................................4

**Figure S2.** Confusion matrices of four machine learning models for 90-day survival prediction in liver failure patients. (A) Logistic regression. (b) Random forest. (C) K-nearest neighbor. (D) Support vector machine. (E) eXtreme Gradient Boosting......4

**Figure S3**. Comparison of ROC curves for the traditional models based on the MELD and Child-Pugh scores illustrating the performance in predicting 90-day survival.......5

**Figure S4**. SHAP diagram for predicting 90-day survival using the logistic regression model. (A): SHAP summary plot of selected features importance. The red part in feature value represents higher value; (B): The importance ranking of the selected features according to the mean (|SHAP value|)..............................................................5

**Figure S5.** ROC curve comparison of logistic regression models for liver failure patients in predicting 90-day survival at different time points. (A) ROC curve based on data from the day before treatment. (B) ROC curve based on data from the day after treatment.................................................................................................................5

Table S1. Predictive Performance of Machine Learning Models for 90-Day Survival in Liver Failure Patients After Artificial Liver Treatment

| Dataset | AUC (95% CI) | Accuracy | Sensitivity | Specificity | PPV | NPV | F1-score | Threshold |
| --- | --- | --- | --- | --- | --- | --- | --- | --- |
| Logistic Regression | 0.884 (0.786 - 0.960) | 0.753 | 0.681 | 1.000 | 1.000 | 0.464 | 0.810 | 0.859 |
| Random Forest | 0.797 (0.663 - 0.914) | 0.583 | 0.468 | 1.000 | 1.000 | 0.342 | 0.638 | 0.860 |
| K-Nearest Neighbors | 0.788 (0.642 - 0.907) | 0.783 | 0.787 | 0.769 | 0.925 | 0.500 | 0.851 | 0.800 |
| Support Vector Machine | 0.732 (0.527 - 0.899) | 0.833 | 0.894 | 0.615 | 0.894 | 0.615 | 0.894 | 0.718 |
| eXtreme Gradient Boosting | 0.769 (0.585 - 0.918) | 0.850 | 0.936 | 0.538 | 0.880 | 0.700 | 0.907 | 0.323 |
| AUC: area under the receiver operating characteristic curve; PPV: positive predictive value; NPV: negative predictive value. | | | | | | | | |

Table S2. The results of LASSO and stepwise logistic regression analysis based on data from the day before and the day after treatment

| Variable | Coefficient | Standard error | z-value | p-value | 95% CI |
| --- | --- | --- | --- | --- | --- |
| The Day Before Treatment | | | | | |
| IBIL | -0.874 | 0.261 | -3.346 | 0.001 | -1.386 to -0.362 |
| Albumin | 0.656 | 0.275 | 2.387 | 0.017 | 0.117 to 1.195 |
| PT | -0.920 | 0.259 | -3.554 | 0.000 | -1.427 to -0.413 |
| CRP | -0.673 | 0.231 | -2.920 | 0.004 | -1.125 to -0.221 |
| Age | -0.504 | 0.257 | -1.961 | 0.050 | -1.008 to -0.000 |
| The Day After Treatment | | | | | |
| PT | -0.755 | 0.225 | -3.449 | 0.001 | -1.215 to -0.335 |
| Age | -0.754 | 0.257 | -2.935 | 0.003 | -1.258 to -0.251 |
| TBIL | -0.797 | 0.231 | -3.457 | 0.001 | -1.250 to -0.345 |
| RBC | 0.738 | 0.249 | 2.962 | 0.003 | 0.250 to 1.226 |
| CI: confidence interval; IBIL: indirect bilirubin; PT: prothrombin time; CRP: C-reactive protein; TBIL: total bilirubin; RBC: red blood cell. | | | | | |

Table S3. Predictive performance of logistic regression models for 90-day survival in liver failure patients at different time points

| Time Point | AUC (95% CI) | Accuracy | Sensitivity | Specificity | PPV | NPV | F1-score | Threshold |
| --- | --- | --- | --- | --- | --- | --- | --- | --- |
| At the time of admission | 0.884 (0.786 - 0.960) | 0.753 | 0.681 | 1.000 | 1.000 | 0.464 | 0.810 | 0.859 |
| The day before treatment | 0.869 (0.747 - 0.970) | 0.850 | 0.872 | 0.769 | 0.932 | 0.625 | 0.901 | 0.761 |
| The day after treatment | 0.859 (0.757- 0.941) | 0.767 | 0.702 | 1.000 | 1.000 | 0.481 | 0.825 | 0.902 |
| AUC: area under the receiver operating characteristic curve; PPV: positive predictive value; NPV: negative predictive value. | | | | | | | | |

Figure S1. Feature selection based on LASSO model. (A) Cross-validation error plot for LASSO regression, showing the mean squared error against log(λ) with the optimal λ values indicated by vertical dashed lines. (B) Plots for LASSO regression coefficients over different values of the penalty parameter.


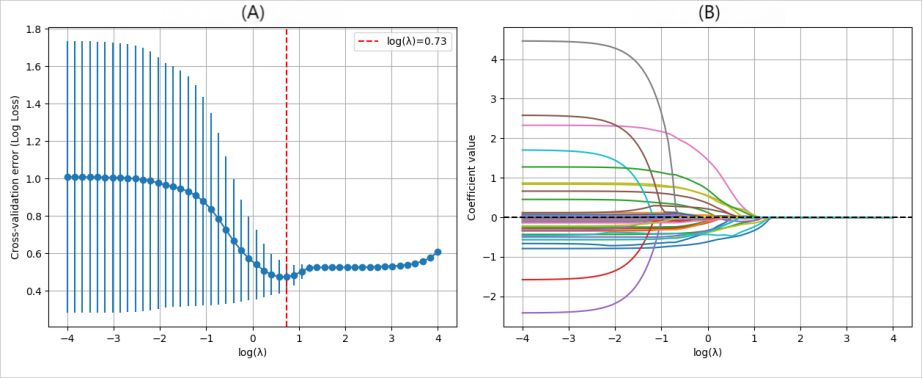


Figure S2. Confusion matrices of four machine learning models for 90-day survival prediction in liver failure patients. (A) Logistic regression. (b) Random forest. (C) K-nearest neighbor. (D) Support vector machine. (E) eXtreme Gradient Boosting.


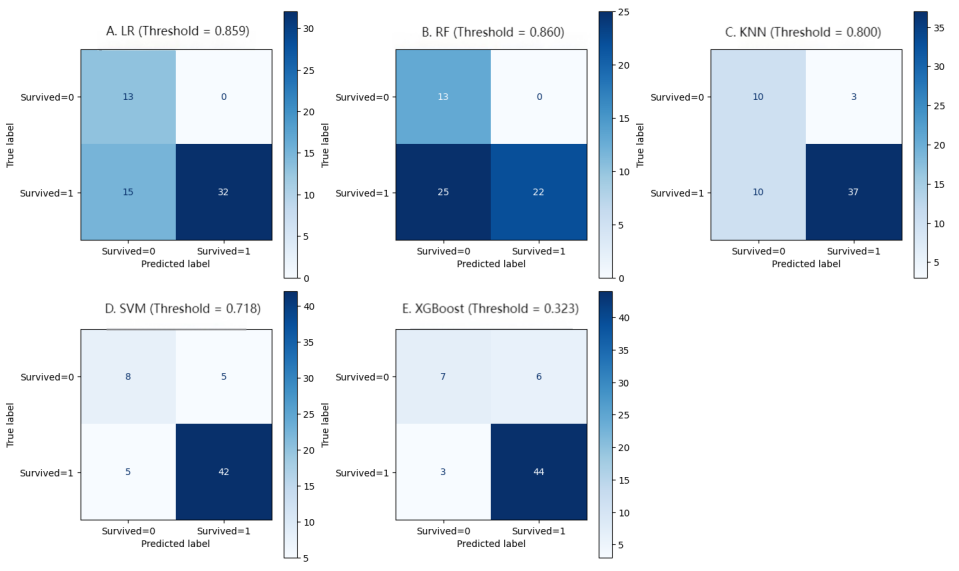


Figure S3. Comparison of ROC curves for the traditional models based on the MELD and Child-Pugh scores illustrating the performance in predicting 90-day survival.


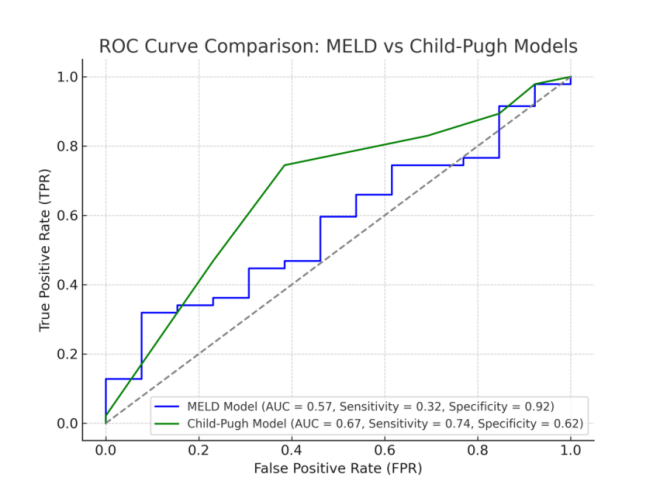


Figure S4. SHAP diagram for predicting 90-day survival using the logistic regression model. (A): SHAP summary plot of selected features importance. The red part in feature value represents higher value; (B): The importance ranking of the selected features according to the mean (|SHAP value|).


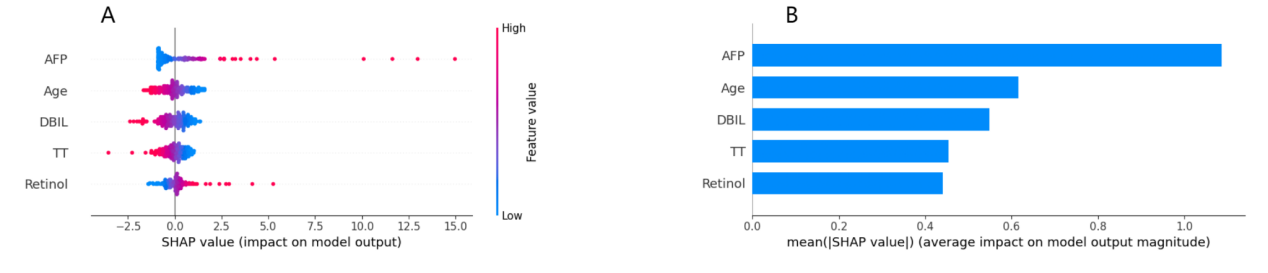


Figure S5. ROC curve comparison of logistic regression models for liver failure patients in predicting 90-day survival at different time points. (A) ROC curve based on data from the day before treatment. (B) ROC curve based on data from the day after treatment.


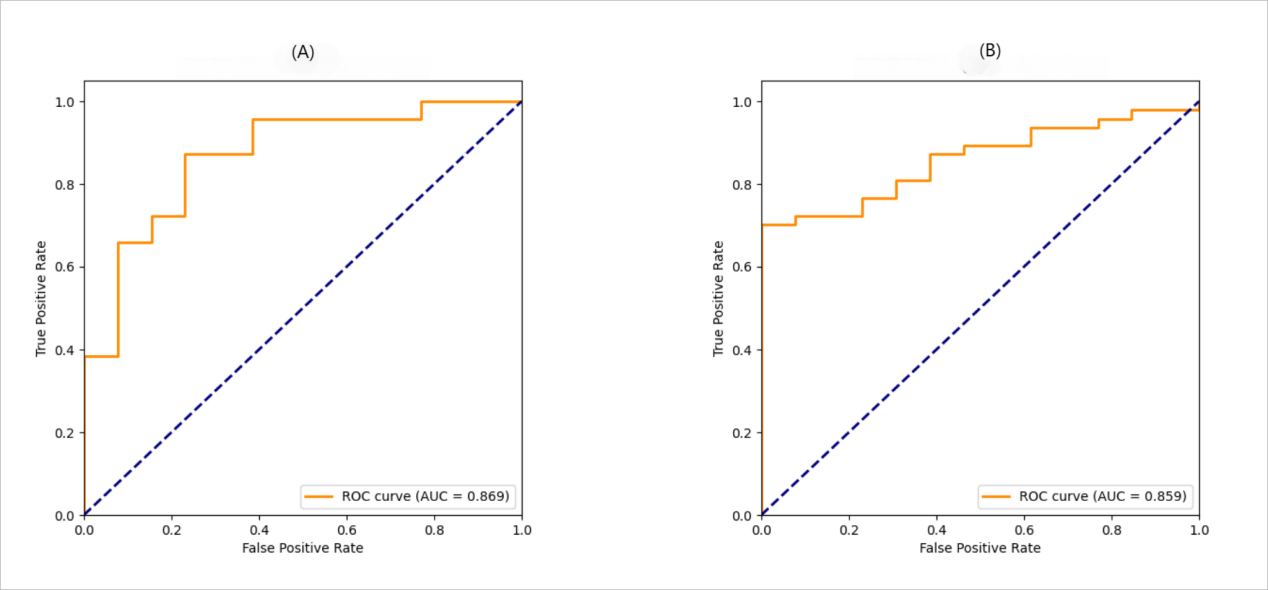

Supplement: Supplementary file 1 [file Supplementaryfile1.docx]
